# Curcumin reinforces MSC‐derived exosomes in attenuating osteoarthritis via modulating the miR‐124/NF‐kB and miR‐143/ROCK1/TLR9 signalling pathways

**DOI:** 10.1111/jcmm.15714

**Published:** 2020-08-09

**Authors:** Bo Qiu, Xiongfeng Xu, Peng Yi, Yarong Hao

**Affiliations:** ^1^ Department of Orthopedics Renmin Hospital of Wuhan University Wuhan China; ^2^ Department of Geriatrics Renmin hospital of Wuhan University Wuhan China

**Keywords:** curcumin, exosome, miR‐124, miR‐143, NF‐kB, osteoarthritis, ROCK1, TLR9

## Abstract

Curcumin treatment was reported to delay the progression of OA, but its underlying mechanism remains unclear. In this study, we aimed to investigate the molecular mechanism underlying the role of curcumin in OA treatment. Accordingly, by conducting MTT and flow cytometry assays, we found that the exosomes derived from curcumin‐treated MSCs helped to maintain the viability while inhibiting the apoptosis of model OA cells. Additionally, quantitative real‐time PCR and Western blot assays showed that the exosomes derived from curcumin‐treated MSCs significantly restored the down‐regulated miR‐143 and miR‐124 expression as well as up‐regulated NF‐kB and ROCK1 expression in OA cells. Mechanistically, curcumin treatment decreased the DNA methylation of miR‐143 and miR‐124 promoters. In addition, the 3’ UTRs of NF‐kB and ROCK1 were proven to contain the binding sites for miR‐143 and miR‐124, respectively. Therefore, the up‐regulation of miR‐143 and miR‐124 in cellular and mouse OA models treated with exosomes remarkably restored the normal expression of NF‐kB and ROCK1. Consequently, the progression of OA was attenuated by the exosomes. Our results clarified the molecular mechanism underlying the therapeutic role of MSC‐derived exosomes in OA treatment.

## 1 INTRODUCTION

Osteoarthritis (OA) is deemed as a disorder triggered by the imbalance between anabolic and catabolic activities of articular joints.^1^ The progression of OA is regulated by matrix metalloproteinases (MMP), including MMP‐3 and MMP‐1, which participated in the degradation of extracellular matrix.^2^ In particular, MMP‐3 and MMP‐1 can be activated by several inflammatory mediators, including IL‐1β and TNF‐α, that are present in joint fluids.^3^ Unfortunately, there is no effective way to prevent OA recurrence. Nevertheless, it was presented that MSC‐derived exosomes could be utilized as a new cell‐free treatment of OA and joint damage.^4^

Mesenchymal stem cells (MSCs) are endogenous stem cells with the ability to differentiate into neurocytes, adipocytes, chondrocytes and osteocytes.^5^ Mesenchymal stem cells have exhibited their therapeutic role in many diseases, including lung cancer,^6^ melanoma and glioblastoma.^7^ The MSCs derived from adipose tissues have been expanded in vitro for transplantation and tissue repair.^8^ As a result, due to their chondrogenic potential and the ability to generate extracellular matrix, MSCs may have a great potential in OA therapy.^9^ In addition, MSCs exert immunomodulatory effects via secreting growth factors and anti‐inflammatory factors, thus alleviating OA‐induced inflammation.^10^

Carrying various macromolecules including DNA, miRNA and mRNA, along with fusion‐promoting surface lipids and proteins,^11^, ^12^ exosomes are released from a wide range of different types of cells.^13^ On the other hand, as a key active component of Curcuma longa containing a β‐dike tone and two phenols, curcumin has been used as a natural drug in wound healing.^14^ Due to its anti‐cancer, anti‐inflammatory, anti‐oxidant and chemotherapeutic features, curcumin is also used to treat various clinical disorders.^15^, ^16^, ^17^ For example, curcumin exerted a therapeutic effect on glioblastoma multiforme cells via affecting the apoptotic, oxidative and inflammatory pathways.^18^ Curcumin also exerts a cardioprotective effect via affecting the levels of inflammation and oxidative stress.^19^, ^20^ Moreover, curcumin was demonstrated to alleviate the symptoms of OA.^21^ Past studies suggested that curcumin can reduce the inflammatory reactions of OA via suppressing the activation of NF‐κB, interleukin 8 (IL‐8), nitric oxide synthase (NOS), prostaglandin E2 (PGE2) and cyclooxygenase‐2 (COX‐2).^22^, ^23^ Also, curcumin plays an anti‐cancer role via targeting regulatory T cells.^24^

MiRNA microarray data demonstrated that curcumin increased miR‐143 expression and altered the expression of miR‐143 targets in the treatment of prostate cancer. It was also shown that miR‐143 expression is reduced in prostate cancer cells, and curcumin exerts an anti‐cancer effect by increasing the expression of miR‐143.^25^, ^26^

The activation of Rho‐associated coiled‐coil containing protein kinase (ROCK) triggers the degradation of cartilages and reduces bone formation. Thus, ROCK may be used as a potential target in OA treatment. The suppression of the Rho/ROCK signalling in chondrocytes can prevent cartilage degradation while stimulating osteoblast mineralization and facilitating bone formation near implants. Furthermore, osteoprotegerin treatment apparently decreases the levels of ROCK2 and ROCK1 to reduce bone resorption. Moreover, ROCK suppression triggers the differentiation of osteoblasts.^27^

NF‐κB plays a critical role in maintaining normal immune and physiological functions.^28^ NF‐κB can also affect the levels of C‐caspase3, Cyto‐c, Bax and Bcl‐2 to induce apoptosis while promoting the production of ADAMTs‐5 and MMP‐13, two factors implicated in the pathogenesis of OA. Moreover, NF‐κB inhibitors were shown to reduce the swelling of joints in rats suffering from arthritis.^29^

It was found that curcumin treatment alleviated the progression of OA.^30^ Furthermore, the administration of curcumin up‐regulated the expression of miR‐143 and miR‐124.^31^, ^32^ ROCK1, a direct target of miR‐124, and its downstream effector TLR9 are implicated in the pathogenesis of OA.^27^ In addition, NF‐kB, a direct target of miR‐124, is also implicated in the development of OA.^33^ In this study, we established cellular and animal models of OA and treated them with EXOs derived from MSCs treated with or without curcumin to investigate the effect of curcumin on the apoptosis of chondrocytes and the signalling pathways of MSCs‐EXO‐CUR/miR‐124/NF‐kB and MSCs‐EXO‐CUR/miR‐143/ROCK1/TLR9.

## 2 MATERIALS AND METHODS

### 2.1 Animal model and treatment

An OA mouse model was established to perform in vivo assessment of the therapeutic effect of curcumin. The mice were acquired from our animal centre and were divided into four groups, that is, a SHAM group, an OA group, an OA + EXO group and an OA + EXO‐CUR group. The mice in the OA + EXO‐CUR group were treated with exosomes derived from MSCs incubated with CUR, while the mice in the OA + EXO group were treated with exosomes derived from MSCs incubated in a CUR‐free medium. In addition, the mice in the SHAM group were subjected to a sham operation and were later treated with PBS only, while the mice in the OA group were subjected to an operation to induce OA. During the experiment, peripheral blood samples and OA tissues or corresponding tissues in sham mice were collected for subsequent analyses. The protocol of various experimental operations in this study was approved by the animal ethics committee of our institute. All operations were carried out in strict accordance with NHC guidelines.

### 2.2 Isolation of MSC‐Exos

After BMSCs reached about 80% confluence, they were rinsed with PBS and incubated in a serum‐free medium for 24 hours. Then, the culture supernatant was harvested via 10 minutes of centrifugation at 300× *g* followed by another round of 15 minutes of centrifugation at 2000× *g*. Then, the supernatant was filtered through a sterile filter with a 0.22 μm aperture (Millipore), followed by 70 minutes of ultra‐filtration at 100 000× *g* to collect exosomes, which were then re‐suspended in PBS. The concentration of proteins in each exosome sample was measured using a BCA kit (Beyotime).

### 2.3 Cell apoptosis analysed via flow cytometry

Chondrocytes were plated with or without 80 μg/mL of BMSC‐Exos into 24‐well plates in a DMEM media containing 10 ng/mL of IL‐1β. After 24 hours of incubation, the apoptotic status of the cells was assessed using flow cytometry.

### 2.4 Bisulphite sequencing

Genomic DNA of tissue samples was separated using an AllPrep mini kit (Qiagen) following the kit instruction. The purity and concentration of isolated genomic DNA were evaluated using a NanoDrop Spectrophotometer (Thermo Fisher Scientific). Then, an EpiTect Bisulfite assay kit (Qiagen) was used to treat the collected genomic DNA samples, and unmethylated and methylated DNA in the samples were quantitated using the COBRA method, followed by direct sequencing and PCR analysis on a PRISM 3100 DNA Analyzer (Applied Biosystems). The status of methylation in the final results was shown as the ratio of methylated CpG in the overall number of analysed CpGs in each sample.

### 2.5 Real‐time PCR

Total RNA was extracted using a Recover All Kit for Total RNA Isolation (Ambion) following the kit instruction. In the next step, a TaqMan kit was used for reverse transcription (Applied Biosystems). Finally, the relative expression of miR‐124, miR‐143, ROCK1 mRNA and NF‐kB mRNA in the samples was quantified using real‐time PCR carried out on a PRISM 7900HT real‐time PCR machine (Applied Biosystems). After real‐time PCR, the obtained results were analysed using SDS 1.4 (Applied Biosystems).

### 2.6 Cell culture and treatment

Primary chondrocytes were purchased from the Cell Bank of Chinese Academy of Sciences (Shanghai, China) and cultured in a DMEM medium (Invitrogen) in 5% CO_2_ at 37°C. Then, the cells were divided into four groups: 1. Control; 2. IL‐1β; 3. IL‐1β + EXO; and 4. IL‐1β + EXO‐CUR. The cells in the control group were treated with PBS. The cells in the IL‐1β group were treated with IL‐1β. The cells in the IL‐1β + EXO group were treated with IL‐1β and EXO collected from MSCs incubated in the absence of curcumin, and the cells in the IL‐1β + EXO‐CUR group were treated with IL‐1β and EXO collected from MSCs incubated in the presence of curcumin. At 48 hours after various treatments, the cells in different groups were collected for subsequent analyses.

### 2.7 Cell proliferation analysis

The proliferation status of cells was assessed using a Cell Counting Kit‐8 (Dojindo) following the kit instruction. During the assay, the absorbance value in each sample well was detected at 450 nm using a plate reader and analysed using SoftMax Pro software (Molecular Devices).

### 2.8 Apoptosis analysis

The apoptosis status of cells was assessed using an Annexin V assay kit purchased from Millipore following the kit instruction.

### 2.9 Western blot analysis

Tissue and cell samples were first lysed for 5 minutes at room temperature in an SDS lysis buffer. Then, the supernatant of lysate was collected via centrifugation. After the amount of protein in each sample was quantified, an equal amount of protein from each sample was boiled and then resolved on a 15% SDS‐PAGE gel. In the next step, the resolved protein was transferred onto nitrocellulose membranes and incubated in sequence with anti‐ROCK1, anti‐TLR9, anti‐NF‐kB monoclonal primary antibodies and appropriate secondary antibodies. All antibodies were acquired from Abcam. After being developed using enhanced chemiluminescence reagents, the protein bands of ROCK1, TLR9 and NF‐kB proteins were imaged and analysed using GAPDH as the internal standard to calculate the relative expression of ROCK1, TLR9 and NF‐kB proteins.

### 2.10 Immunohistochemistry

Fixed mouse tissue sections were treated at 4°C overnight with anti‐NF‐kB primary antibody, rinsed with PBS, and then treated at 37°C for 2 hours with HRP‐conjugated IgG secondary antibodies (1:1000 dilution, Invitrogen). After counter staining with DAPI, the images of sample sections were acquired using an IX71 inverted microscope and analysed.

### 2.11 Vector construction, mutagenesis and luciferase assay

To study the effect of CUR on the transcription efficiency of miR‐134 and miR‐124 promoters, the full length of miR‐134 and miR‐124 promoters were inserted into pcDNA luciferase vectors (Promega). Then, primary chondrocytes were divided into three groups: a Control group, a 1 µmol/L CUR group, and a 5 µmol/L CUR group. After primary chondrocytes in 96‐well plates reached 80% confluency, they were transfected with vectors carrying miR‐134 or miR‐124, and then treated with PBS, 1 µmol/L CUR or 5 µmol/L CUR, respectively. At 48 hours after the start of cell treatment, the luciferase activity of each well was measured on a plate reader using a Bright‐Glo luciferase assay (Promega). Similarly, to validate the target genes of miR‐134 and miR‐124, luciferase vectors containing wild type NF‐kB promoter, a potential target of miR‐134 and miR‐124, and wild type ROCK1 promoter, a potential target of miR‐124, were created. Then, site‐directed mutagenesis was carried out using a Site Directed Mutagenesis Kit (Stratagene) at the miR‐124/miR‐143 binding sites of NF‐kB and ROCK1 promoters to generate mutant sequences of NF‐kB and ROCK1 promoters, which were also inserted into pcDNA luciferase vectors to create vectors of mutant NF‐kB and ROCK1 promoters. Then, primary chondrocytes were plated in 96‐well plates and co‐transfected with miR‐134/miR‐124 mimics or inhibitors in conjunction with mutant or wild type NF‐kB and ROCK1 plasmids. At 48 hours after the start of transfection, the luciferase activity of each well was measured on a plate reader using the Bright‐Glo luciferase assay (Promega).

### 2.12 TUNEL assay

The apoptotic status of tissue samples collected from the four groups of mice was analysed using a TUNEL assay kit (Thermo Fisher Scientific) following the kit instruction.

### 2.13 Statistical analysis

SPSS 15.0 software (SPSS) was utilized to carry out all statistical analyses. Student's *t* tests were utilized to determine the statistical significance (α = 0.05) of differences among different groups of samples.

## 3 RESULTS

### 3.1 Exosomes derived from curcumin‐treated MSCs maintained the viability of primary chondrocytes

**Figure 1:**
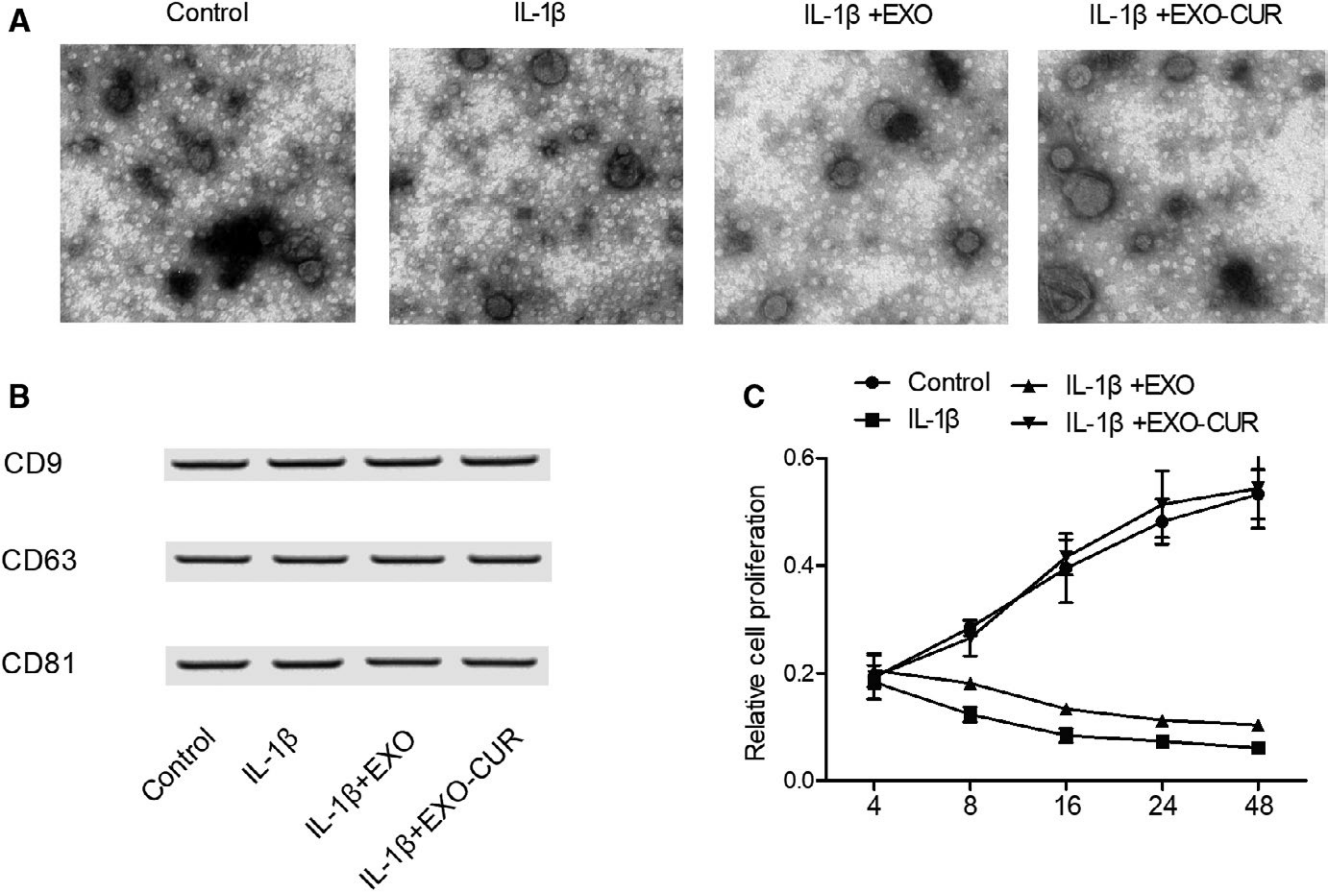
Characterization of exosomes derived from MSCs as well as the viability of primary chondrocytes treated under different conditions. A, Morphology of exosomes derived from MSCs. B, Analysis of exosome‐specific CD9, CD63 and CD81 proteins with Western blot. C, The viability of primary chondrocytes was the highest when the cells were treated with exosomes derived from curcumin‐treated MSCs, and the viability of primary chondrocytes was the lowest when the cells were treated with IL‐1β

Examined using transmission electron microscopy, the exosomes derived from MSCs appeared as round‐shaped bubbles ranging from 50‐150 nm in size (Figure 1A). The identity of exosomes was further checked with Western blot to verify the expression of exosome‐specific protein markers CD9, CD63 and CD81 (Figure 1B). As shown in Figure 1C, MTT results showed that the exosomes derived from MSCs could partially restore the viability of primary chondrocytes decreased by IL‐1β. Moreover, the exosomes derived from curcumin‐treated MSCs fully restored the viability of primary chondrocytes decreased by IL‐1β.

### 3.2 Exosomes derived from curcumin‐treated MSCs protected primary chondrocytes against IL‐1β‐induced apoptosis

**Figure 2:**
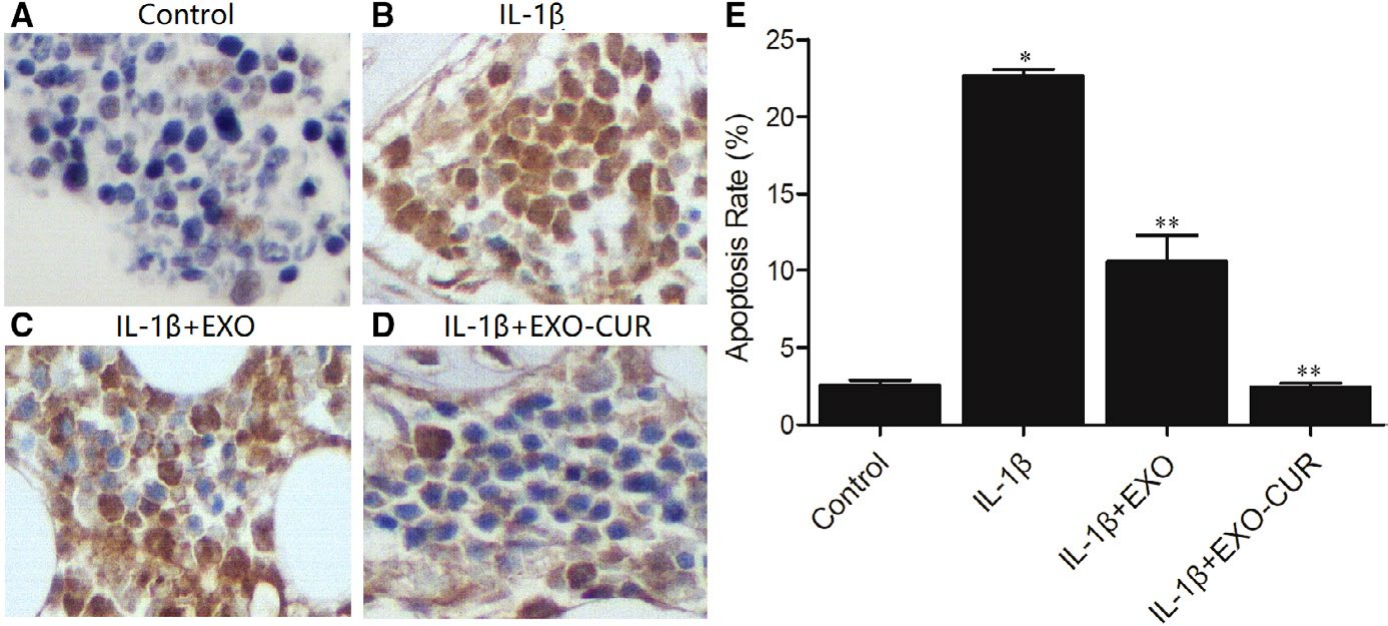
Exosomes derived from curcumin‐treated MSCs decreased IL‐1β‐induced apoptosis. A‐D, The result of TUNEL assay showed the apoptosis of primary chondrocytes treated under different conditions: control (A), IL‐1β(B), IL‐1β + EXO (C), IL‐1β + EXO‐CUR (D). E, The apoptosis of primary chondrocytes was increased when treated with IL‐1β, while the treatment with exosomes derived from curcumin‐treated MSCs dramatically reduced apoptosis (* *P* value < .05 vs control group, ** *P* < .05 vs IL‐1β group)

Hoechst 33 342 staining was carried out to assess the apoptotic status of primary chondrocytes treated by IL‐1β. Then, the cells were treated with exosomes derived from curcumin‐treated MSCs. IL‐1β (Figure 2B,E) caused obvious apoptosis of primary chondrocytes when compared with the control group (Figure 2A,E), while the exosomes derived from MSCs reduced the apoptosis to a certain degree (Figure 2C,E). Moreover, the exosomes derived from curcumin‐treated MSCs almost fully inhibited IL‐1β‐induced apoptosis (Figure 2D,E).

### 3.3 Exosomes derived from curcumin‐treated MSCs restored the expression of miRNAs and genes related to OA

**Figure 3:**
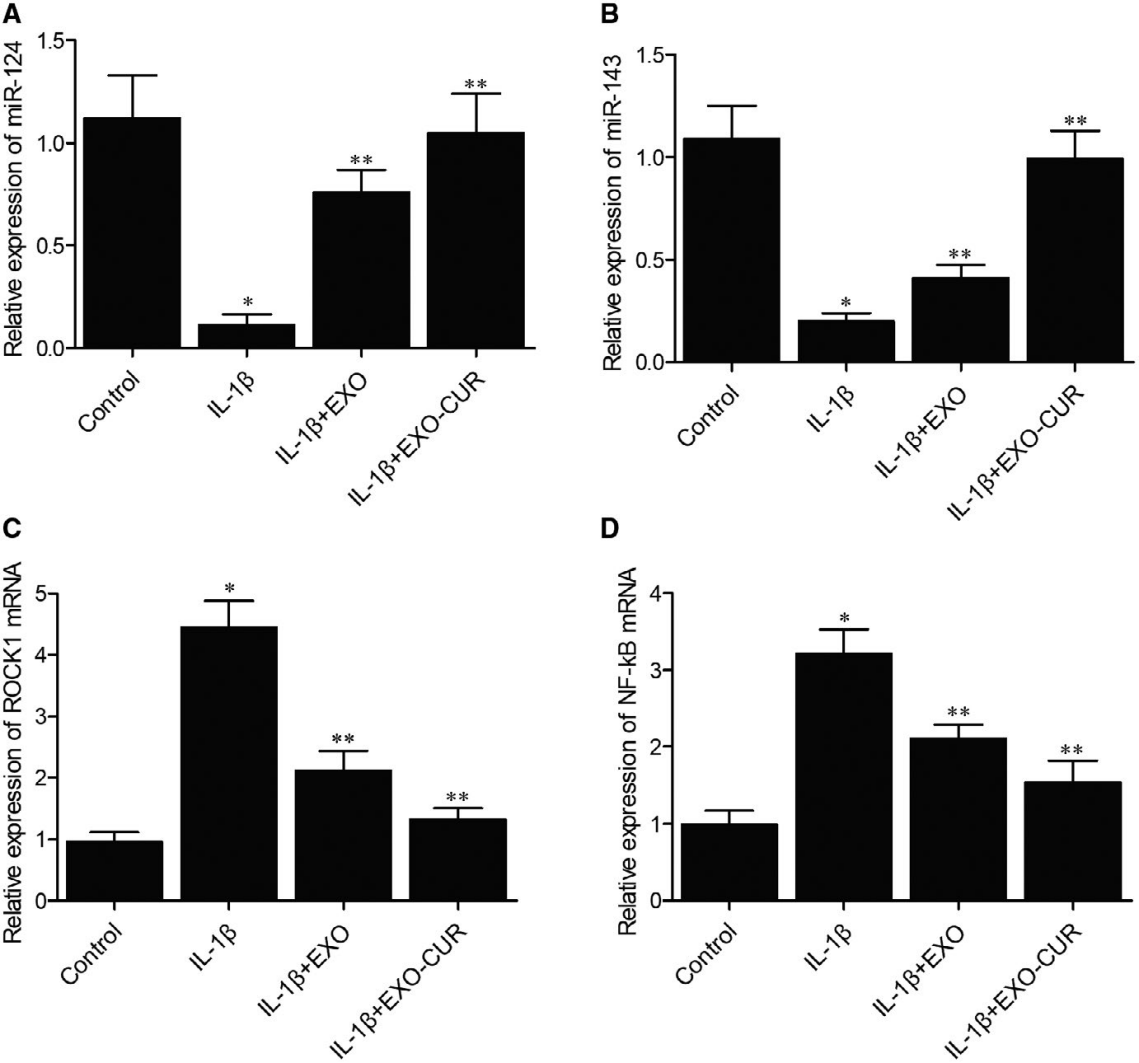
Exosomes derived from curcumin‐treated MSCs recovered the normal expression of miR‐124, miR‐143, ROCK1 mRNA and NF‐kB mRNA after IL‐1β treatment (*P < .05 vs control group, **P < .05 vs IL‐1β group). A‐B, IL‐1β decreased the expression of miR‐124 (A) and miR‐143 (B), while the expression of miR‐124 and miR‐143 was fully restored by exosomes derived from curcumin‐treated MSCs. C‐D, IL‐1β elevated the expression of ROCK1 (C) and NF‐kB (D) mRNAs, whose expression was reduced by exosomes derived from curcumin‐treated MSCs

**Figure 4:**
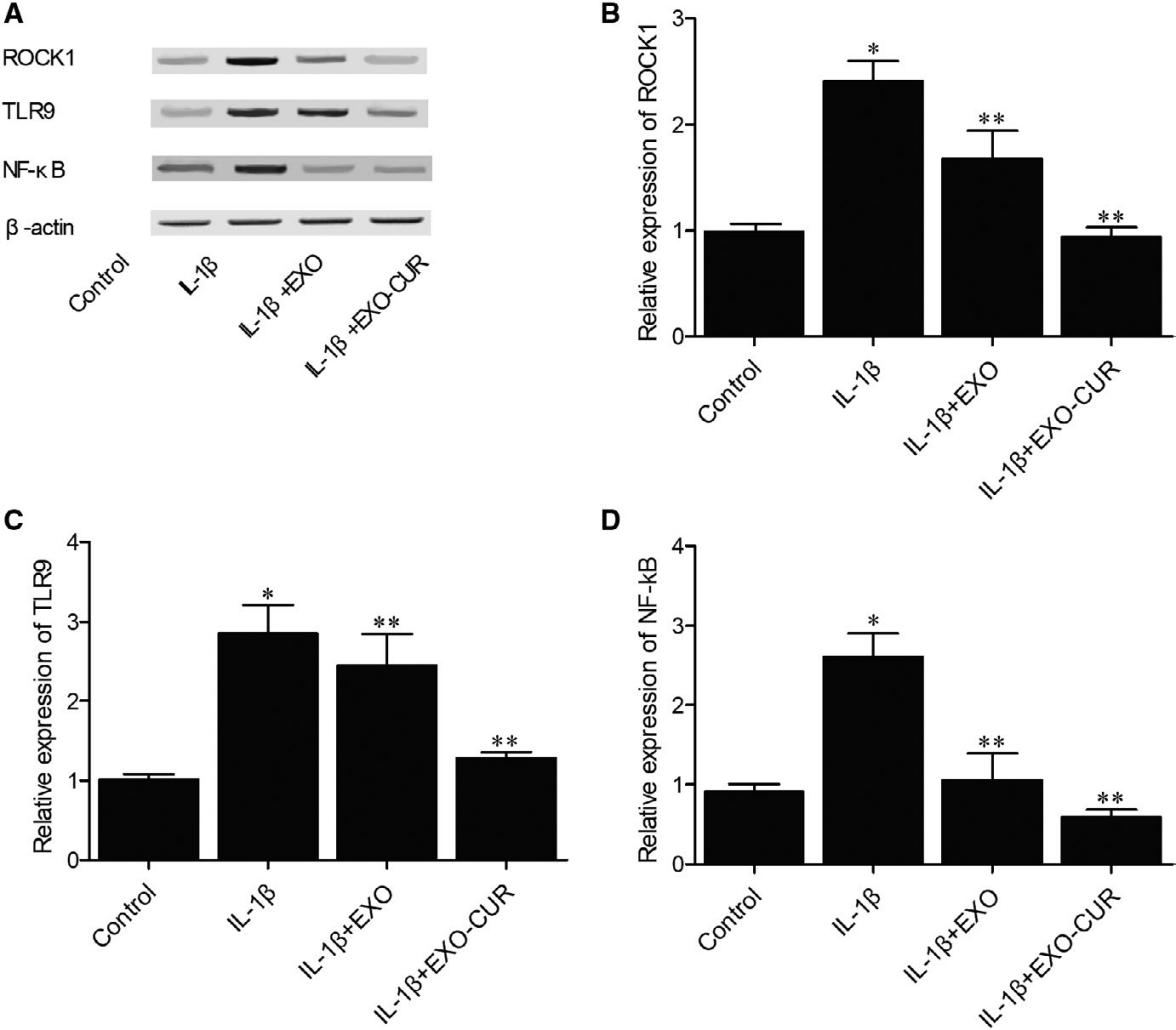
Exosomes derived from curcumin‐treated MSCs recovered the normal expression of ROCK1, TLR9 and NF‐kB proteins in cells treated with IL‐1β (*P < .05 vs control group, **P < .05 vs IL‐1β group). A, In primary chondrocytes treated with IL‐1β, the Western blot analysis showed abnormally increased expression of ROCK1, TLR9 and NF‐kB proteins, whose expression was suppressed by exosomes derived from curcumin‐treated MSCs. B‐D, Primary chondrocytes treated with IL‐1β displayed increased expression of ROCK1 (B), TLR9 (C) and NF‐kB (D) proteins, whose expression was dramatically inhibited by exosomes derived from curcumin‐treated MSCs

Quantitative real‐time PCR was performed to detect the expression of miR‐143, miR‐124, ROCK1 and NF‐kB in the cellular model of OA. Primary chondrocytes treated with IL‐1β showed declined expression of miR‐124 (Figure 3A) and miR‐143 (Figure 3B). When exosomes derived from MSCs were added into the media, the expression of both miRNAs was increased to a certain extent, albeit at a level still lower than that of the control. However, when the cells were treated with exosomes derived from curcumin‐treated MSCs, the expression of miRNAs was almost fully restored. On the contrary, abnormally high expression of ROCK1 (Figure 3C) and NF‐kB (Figure 3D) mRNAs was seen in primary chondrocytes treated with IL‐1β, while the treatment with exosomes derived from MSCs could decrease the expression of ROCK1 and NF‐kB mRNAs to a certain degree. In addition, the exosomes derived from curcumin‐treated MSCs showed a higher efficacy. Moreover, Western blot further confirmed the above changes in the protein expression of ROCK1, TLR9 and NF‐kB. As can be seen, the protein expression of ROCK1 (Figure 4A), TLR9 (Figure 4B) and NF‐kB (Figure 4C) was up‐regulated by IL‐1β and suppressed by exosomes derived from MSCs treated with or without curcumin.

### 3.4 Curcumin up‐regulated miR‐143 and miR‐124 expression by reducing the DNA methylation of their promoters

**Figure 5:**
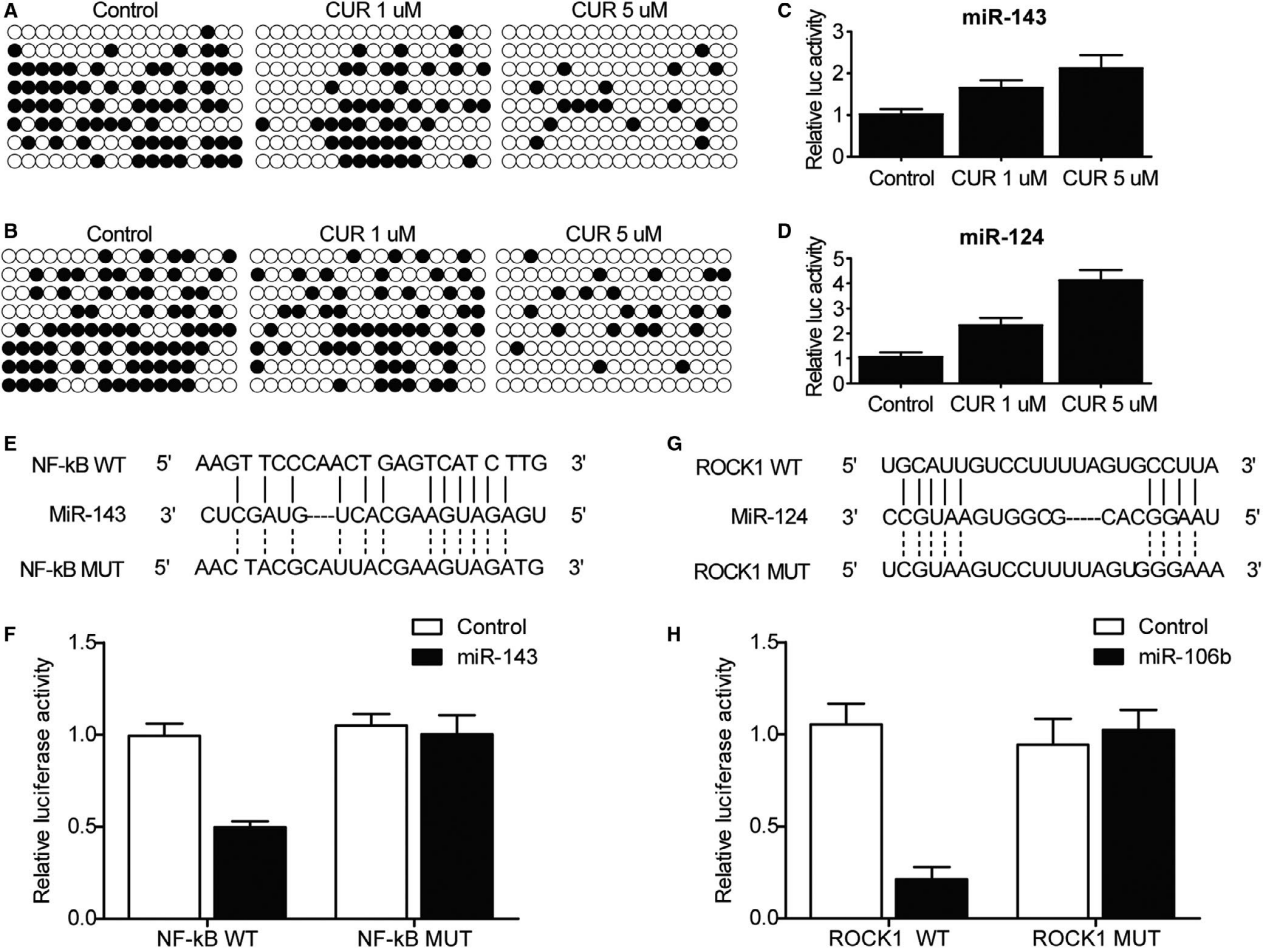
Curcumin up‐regulated the expression of miR‐143 and miR‐124, which subsequently inhibited NF‐kB and ROCK1 expression, respectively. A‐B, Bisulphite sequencing showed reduced DNA methylation of miR‐143(A) and miR‐124(B) promoters in primary chondrocytes treated with a higher concentration of curcumin. C‐D, Luciferase assay showed an elevated transcription efficiency of miR‐143(C) and miR‐124(D) promoters in primary chondrocytes treated with a higher concentration of curcumin. E, Potential target site of miR‐143 on NF‐kB 3’ UTR. F, The luciferase activity of WT NF‐kB vector was inhibited by miR‐143. G, Potential target site of miR‐124 on ROCK1 3’ UTR. H, The luciferase activity of WT ROCK1 vector was inhibited by miR‐124

To gain a full insight into the mechanism underlying the therapeutic effect of curcumin on OA, bisulphite sequencing was used to check the DNA methylation status of the promoters of miR‐143 and miR‐124 in primary chondrocytes treated with 1 and 5 µmol/L of curcumin. The results demonstrated that curcumin treatment clearly decreased the DNA methylation of miR‐143 (Figure 5A) and miR‐124 (Figure 5B) promoters. Since DNA methylation in promoter regions usually inhibits gene transcription, curcumin‐induced reduction in DNA methylation exerted a positive effect on miR‐143 (Figure 5C) and miR‐124 (Figure 5D) expression. There was a miR‐143 binding site in the 3’ UTR of NF‐kB (Figure 5E), and the luciferase activity of NF‐kB 3’ UTR was remarkably inhibited by wild type miR‐143 (Figure 5F). Similarly, miR‐124 inhibited the luciferase activity of ROCK1 3’ UTR (Figure 5G,H).

### 3.5 The apoptosis of chondrocytes was reduced by exosomes derived from curcumin‐treated MSCs

**Figure 6:**
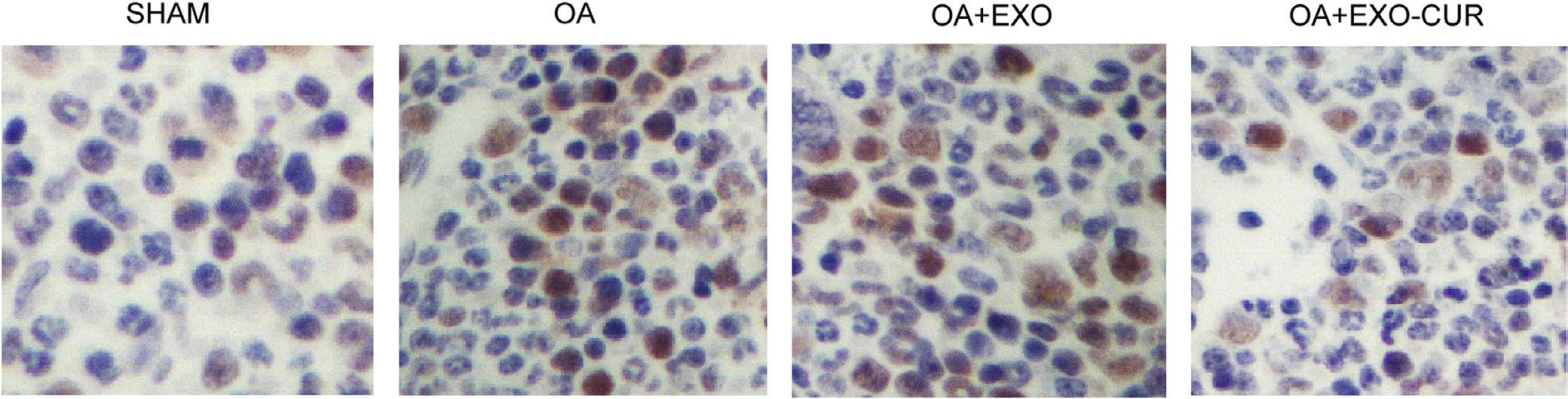
The TUNEL analysis demonstrated that chondrocyte apoptosis in OA mice was reduced by exosomes derived from curcumin‐treated MSCs

As shown in Figure 6, the TUNEL analysis revealed a high level of chondrocyte apoptosis in OA mice, while the treatment with exosomes derived from curcumin‐treated MSCs suppressed chondrocyte apoptosis.

### 3.6 Exosomes derived from curcumin‐treated MSCs restored the expression of miRNAs and genes related to OA

**Figure 7:**
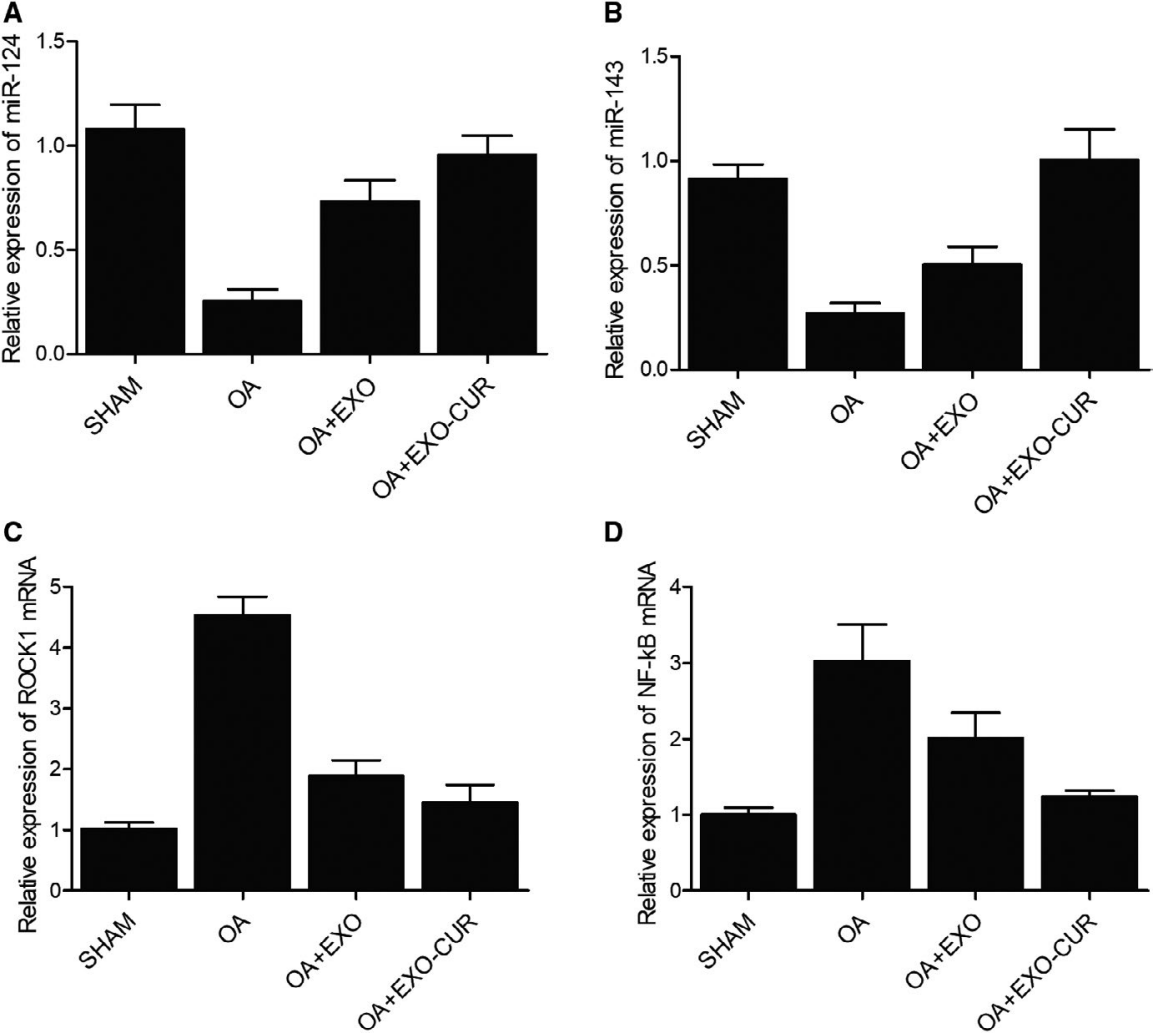
The exosomes derived from curcumin‐treated MSCs recovered the normal expression of miR‐124, miR‐143, ROCK1 mRNA and NF‐kB mRNA in OA mice. A‐B, IL‐1β‐induced decrease in the expression of miR‐124(A) and miR‐143(B) was fully restored by exosomes derived from curcumin‐treated MSCs. C‐D, IL‐1β‐induced increase in the expression of ROCK1(C) and NF‐kB(D) mRNAs was reduced by exosomes derived from curcumin‐treated MSCs

**Figure 8:**
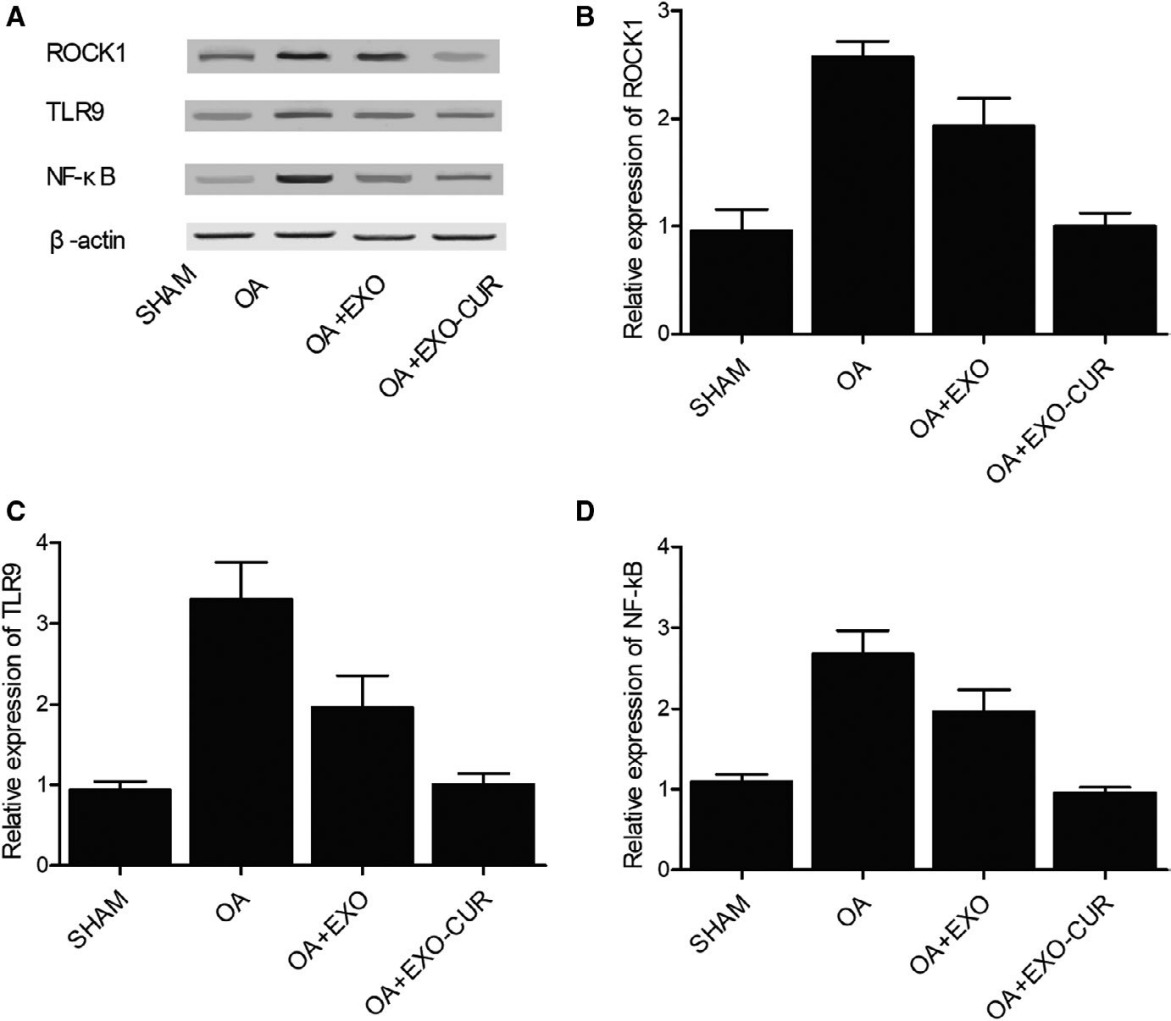
Exosomes derived from curcumin‐treated MSCs recovered the normal expression of ROCK1, TLR9 and NF‐kB proteins in OA mice. A, Western blot analysis showed abnormally increased ROCK1, TLR9 and NF‐kB protein expression in OA mice, while the exosomes derived from curcumin‐treated MSCs recovered the normal expression of ROCK1, TLR9 and NF‐kB proteins. B‐D, The expression of ROCK1(B), TLR9(C) and NF‐kB(D) proteins was increased in OA mice, while the exosomes derived from curcumin‐treated MSCs recovered the normal expression of ROCK1, TLR9 and NF‐kB proteins

**Figure 9:**
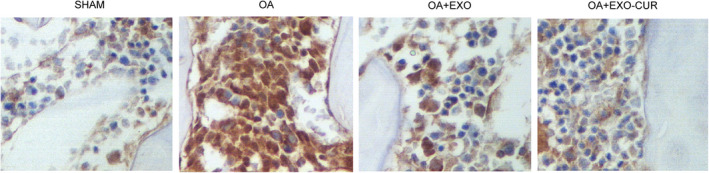
Immunohistochemistry results showed that the exosomes derived from curcumin‐treated MSCs recovered the normal expression of ROCK1 protein in OA mice. A, Immunohistochemistry analysis showed abnormally increased NF‐kB protein expression in OA mice, while the exosomes derived from curcumin‐treated MSCs recovered the normal expression of NF‐kB protein. B, Immunohistochemistry analysis showed abnormally increased ROCK1 protein expression in OA mice, while the exosomes derived from curcumin‐treated MSCs recovered the normal expression of ROCK1 protein

Then, an OA mouse model was established to assess the in vivo effect of curcumin. Similar to the results obtained from the cellular model, the OA mouse model showed declined expression of miR‐124 (Figure 7A) and miR‐143 (Figure 7B) along with elevated expression of ROCK1 (Figure 7C) and NF‐kB (Figure 7D). In addition, the treatment with exosomes showed similar therapeutic effects as those observed in the cellular model. Similarly, Western blot (Figure 8) and IHC results (Figure 9) also confirmed the changes in the protein expression of ROCK1 (Figure 8A,B) and (Figure 9), NF‐kB (Figure 8A,D) and TLR9 (Figure 8A,C) in OA mice.

### 3.7 Schematic description of the effect of MSCs‐EXO‐CUR on the miR‐124/NF‐kB and miR‐143/ROCK1/TLR9 signalling pathways

**Figure 10:**
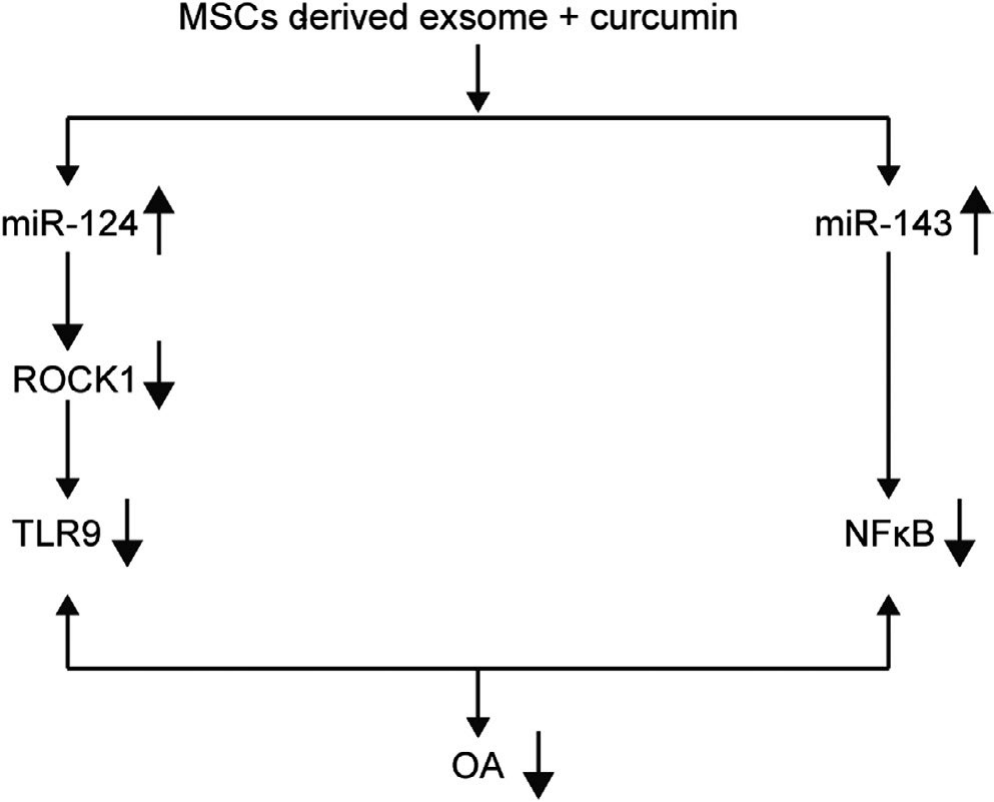
Schematic description of the effect of MSCs‐EXO‐CUR on the miR‐124/NF‐kB and miR‐143/ROCK1/TLR9 signalling pathways

This study demonstrated that curcumin treatment decreased the DNA methylation of miR‐143 and miR‐124 promoters. As a result, miR‐143 and miR‐124 were up‐regulated to further inhibit the expression of their target genes ROCK1 and NF‐kB, which were closely related to the development of OA (Figure 10). In summary, our results provided the mechanistic evidence demonstrating how curcumin treatment delays the progression of OA.

## 4 DISCUSSION

In this study, we established an OA mouse model and performed a TUNEL assay to check the apoptosis status of chondrocytes treated with exosomes derived from MSCs pre‐treated with or without curcumin. The exosomes derived from curcumin‐treated MSCs showed an excellent therapeutic effect on OA mice. In addition, we also showed that the exosomes derived from curcumin‐treated MSCs significantly restored the down‐regulated miR‐143 and miR‐124 expression as well as up‐regulated NF‐kB and ROCK1 expression in OA chondrocytes which might be molecular mechanism underlying the therapeutic effect of MSCs and CUR. Past data showed that MSCs released MSC‐EXOs to ameliorate OA by inhibiting the apoptosis of osteocytes and by elevating the production of bone matrix.^34^ Another studies also demonstrated that milk‐derived EXOs are internalized via various active pathways. It was also demonstrated that exosomal CUR (ExoCUR: exosome isolated from MSC pre‐treated with CUR) is stable during long‐term storage, while the oral administration of ExoCUR apparently results in a higher tissue level of CUR as compared to the oral administration of free CUR. Moreover, the efficacy of ExoCUR is higher in terms of its anti‐apoptotic and anti‐inflammatory performance.^35^ In this study, we collected exosomes from MSCs and used the MTT assay to evaluate the viability of OA cells treated with exosomes. The exosomes derived from curcumin‐treated MSCs showed a considerable therapeutic effect on OA. In addition, we carried out TUNEL assays to assess the apoptosis of OA cells, whose apoptosis was obviously inhibited when they were treated with exosomes derived from curcumin‐treated MSCs. Those results are in line with the previous study as described above in the ability of MSCs derived EXOs and CUR to suppress the apoptosis and promote proliferation of chondrocytes. Our study combined the EXOs and CUR to further promote the therapeutic effect and meanwhile, we also explore the possible molecular mechanism underlying this effect.

It has been shown that the administration of curcumin up‐regulated the expression of miR‐143 and miR‐124 by suppressing methylation of the promoter regions of both miRNAs.^31^, ^32^ ROCK1, a direct target of miR‐124, and its downstream effector TLR9 are implicated in the pathogenesis of OA.^27^ In addition, NF‐kB, a direct target of miR‐124, is also implicated in the development of OA.^33^ Based on the above literature, we hypothesized that curcumin exerted its effect on the ROCK1/TLR9 and NF‐kB via regulating expression of miR‐143 and miR‐124, respectively. In the present study, we used qPCR and Western blot to measure the expression of miR‐143, miR‐124, ROCK1 and NF‐kB and TLR9, respectively. The down‐regulation of miR‐143 and miR‐124 has been reported in the pathogenesis of OA, while the EXOs derived from CUR‐treated MSCs increased the expression of miR‐143 and miR‐124. Meanwhile, the up‐regulation of ROCK1, TLR9 and NF‐kB was also noted in OA, while the EXOs derived from CUR‐treated MSCs decreased the expression of ROCK1, TLR9 and NF‐kB. Furthermore, we showed that CUR decreased the DNA methylation of miR‐143 and miR‐124 promoters to activate their transcription, subsequently inhibiting the expression of their target genes NF‐kB and ROCK1.

ROCK has two isoforms in mammals, that is, ROCK2 and ROCK1. ROCK1 was implicated in the metastasis and invasion of tumours. Majority of previous literature about ROCK1 is about its role in tumorigenesis; for example, one previous research showed that a high level of ROCK1 expression leads to poor tumour differentiation and reduced survival. The ROCK1 signalling is also implicated in tumour cell infiltration and metastasis.^36^ IN addition, ROCK1 is also implicated in tumour cell migration and invasion "Integrated genomic characterization of oesophageal carcinoma", 2017.^37^, ^38^. Additionally, it has been shown that treatment with sevoflurane can reduce ROCK1 expression by elevating miR‐124 expression, and further suppressing MMP‐9 and MMP‐2 expression. Furthermore, the suppression of miR‐124 increases the levels of MMP‐9, MMP‐2 and ROCK1 in the presence of sevoflurane (Gao, Shen, Meng, & He, 2019). In this study, we performed computational analysis and luciferase assay to furthermore confirm the association between miR‐124 and ROCK1 and such association was also confirmed in the cellular and animal model of OA. Together with this, TLR9, as a downstream effector of ROCK1, was also regulated by miR‐124. The activation of TLR‐9 signalling can activate the transcription of NF‐κB and the synthesis of pro‐inflammatory cytokines such as TNF‐α and IL‐6, which in turn trigger the cartilage degradation during OA.^39^, ^40^, ^41^ Macrophages were shown to induce the onset of OA in the presence of bacterial DNAs.^42^ These data suggested that TLR‐9 can affect OA development. Consistent with this, a study of 503 OA patients and 428 controls from Taiwan showed that the risk of OA in the Chinese population was affected by the T allele or TT genotype of the rs187084 polymorphism in TLR‐9.^43^

The transduction of NF‐κB signalling is implicated in the progression of lung cancer, colorectal cancer and liver cancer.^44^, ^45^, ^46^ In addition, NF‐κB expression in SMMC‐7721 liver carcinoma cells was apparently reduced upon the over‐expression of miR‐143, leading to significant suppression in the proliferation of liver carcinoma cells.^32^ The NF‐κB signalling is implicated in OA pathogenesis by regulating the expression of inflammatory factors.^47^, ^48^ Under normal conditions, the NF‐κB in the cytoplasm remains inactive and binds to IκBα, an inhibitory protein. Upon activation by inflammatory factors including IL‐1β, NF‐κB translocates to the nucleus to up‐regulate the expression of genes related to inflammation, such as MMPs, PGE2 COX‐2, iNOS and NO, so as to promote the production of cartilage factors and the death of chondrocytes in OA.^49^ Thus, targeted suppression of NF‐κB may help the treatment of OA. Past reports demonstrated that siRNAs specific to NF‐κB and p65 reduced the levels of MMP‐9, iNOs and COX‐2 in chondrocytes activated by IL‐1β.^50^ In addition, baicalin apparently suppressed IκBαin, NF‐κB and p65 phosphorylation while delaying IκBαin degradation in chondrocytes activated by IL‐1β. Moreover, baicalin apparently reduced the activation of NF‐kB promoter in the presence of IL‐1β.^51^

## 5 CONCLUSION

In this study, we treated cellular and animal models of OA with EXOs derived from MSCs treated with or without curcumin to investigate the effect of curcumin on the apoptosis of chondrocytes. We confirmed that curcumin was effective in slowing OA progression. In addition, curcumin may exert its effects by regulating the miR‐124/NF‐kB and miR‐143/ROCK1/TLR9 pathways.

## CONFLICT OF INTERESTS

The authors declare that they have no competing interests.

## AUTHOR CONTRIBUTION

**Bo Qiu:** Conceptualization (equal); Investigation (equal); Project administration (equal); Supervision (equal); Writing‐original draft (equal). **Xiongfeng Xu:** Formal analysis (equal); Investigation (equal); Resources (equal); Visualization (equal). **Peng Yi:** Investigation (equal); Software (equal); Visualization (equal). **Yarong Hao:** Conceptualization (equal); Investigation (equal); Project administration (equal); Supervision (equal); Writing‐original draft (equal).

## Data Availability

The data that support the findings of this study are available from the corresponding author upon request.
